# Supplementary figures and images for: TLNRD1 is a CCM complex component and regulates endothelial barrier integrity
Source: J Cell Biol. 2024 Jul 16;223(9):e202310030. doi: 10.1083/jcb.202310030 (PMC11252447; doi:10.1083/jcb.202310030)

Figure 1D

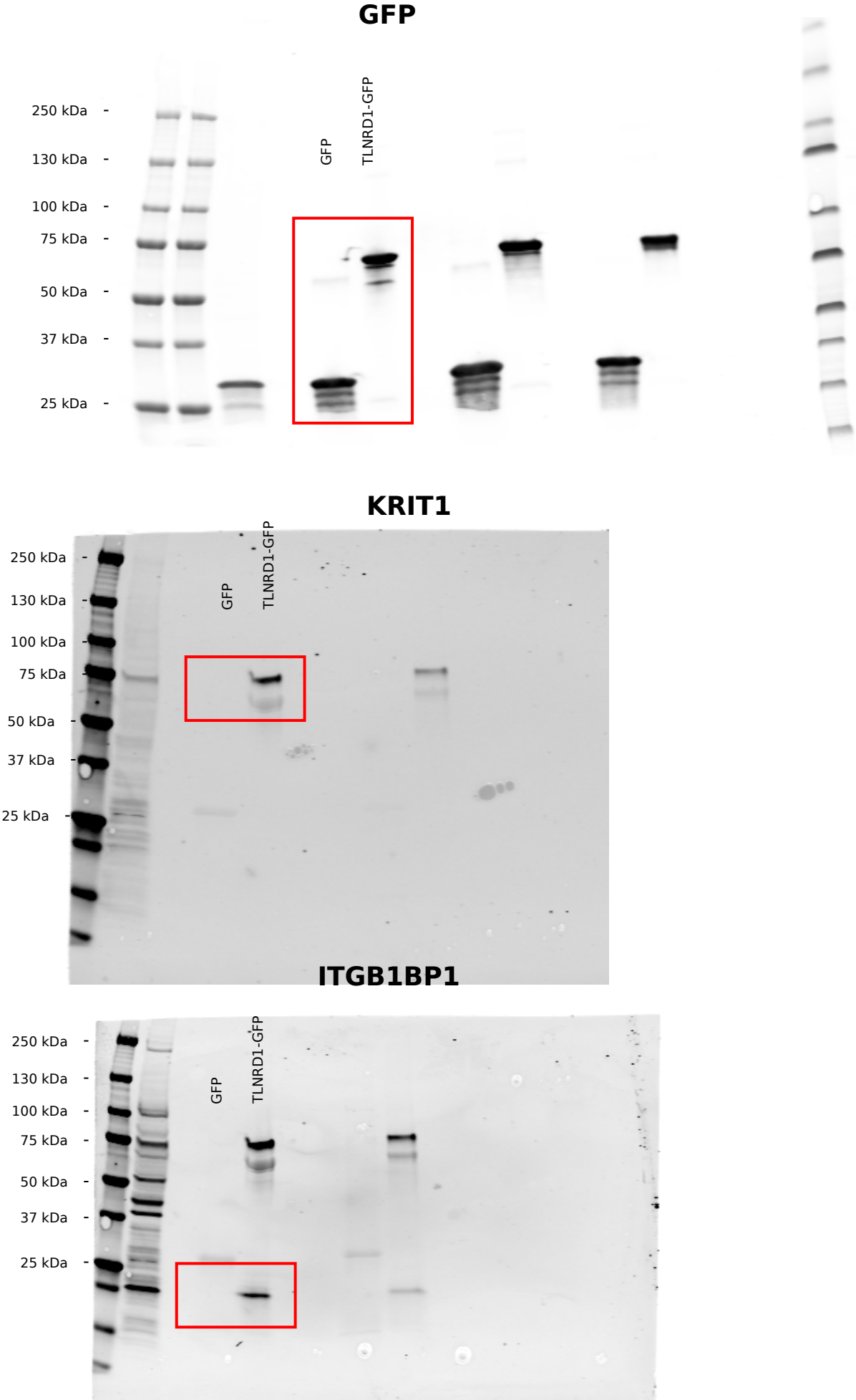

Supplement: SourceData F1 — is the source file for Fig. 1. [file JCB_202310030_SourceDataF1.pdf]

Figure 4D

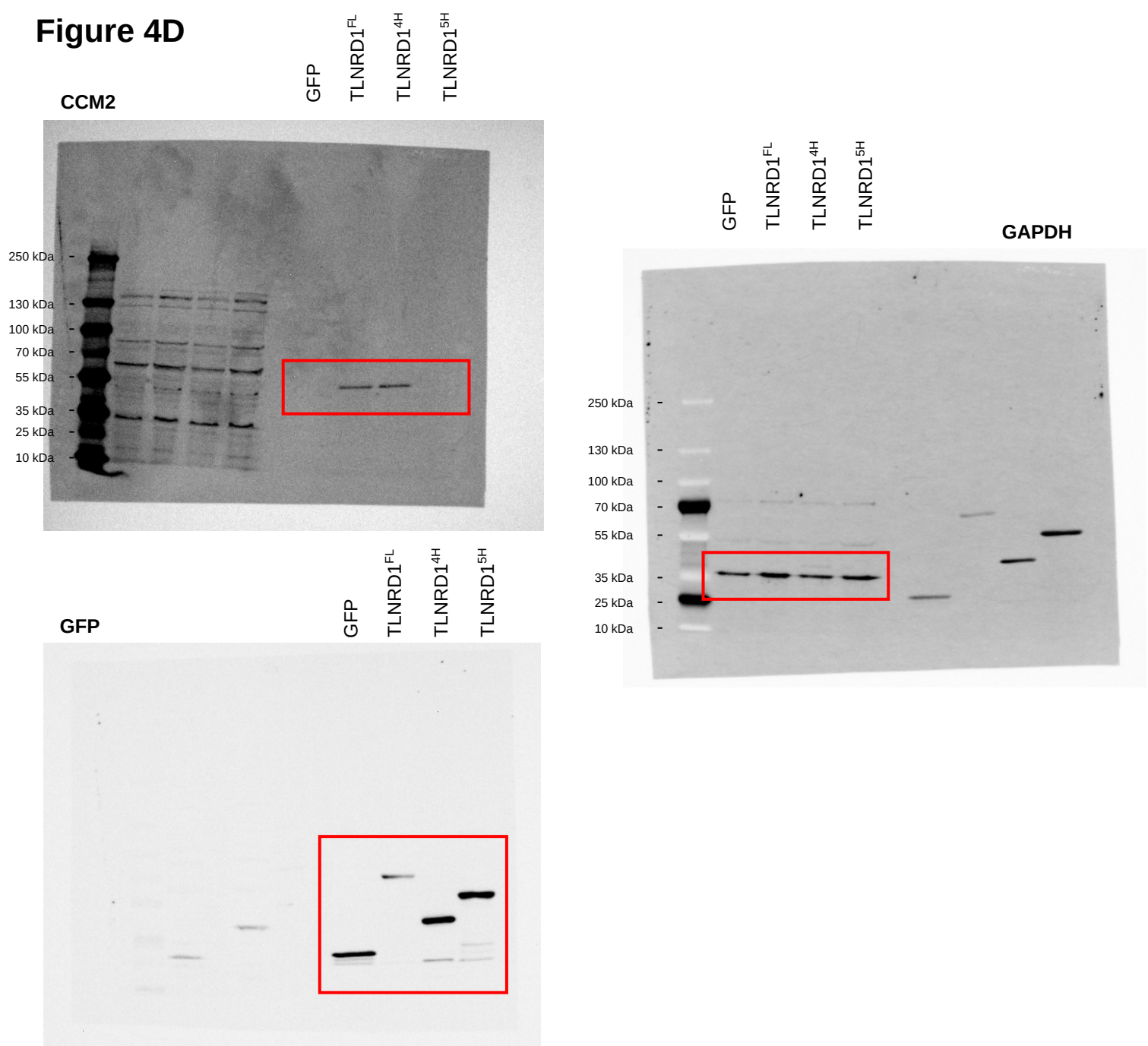

Figure 4F

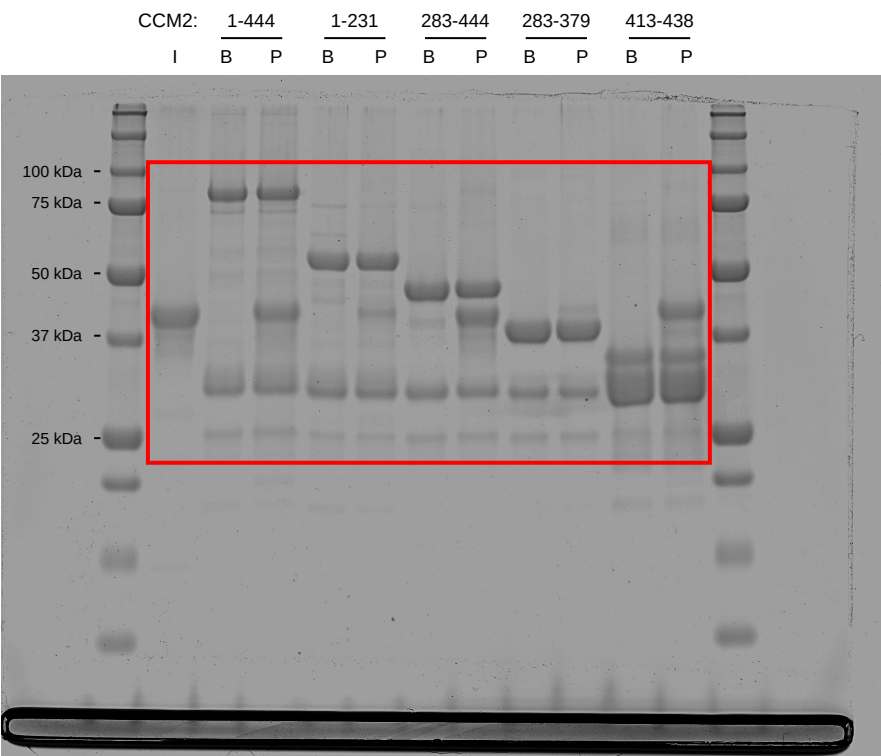

Supplement: SourceData F4 — is the source file for Fig. 4. [file JCB_202310030_SourceDataF4.pdf]

Figure 7D

WT

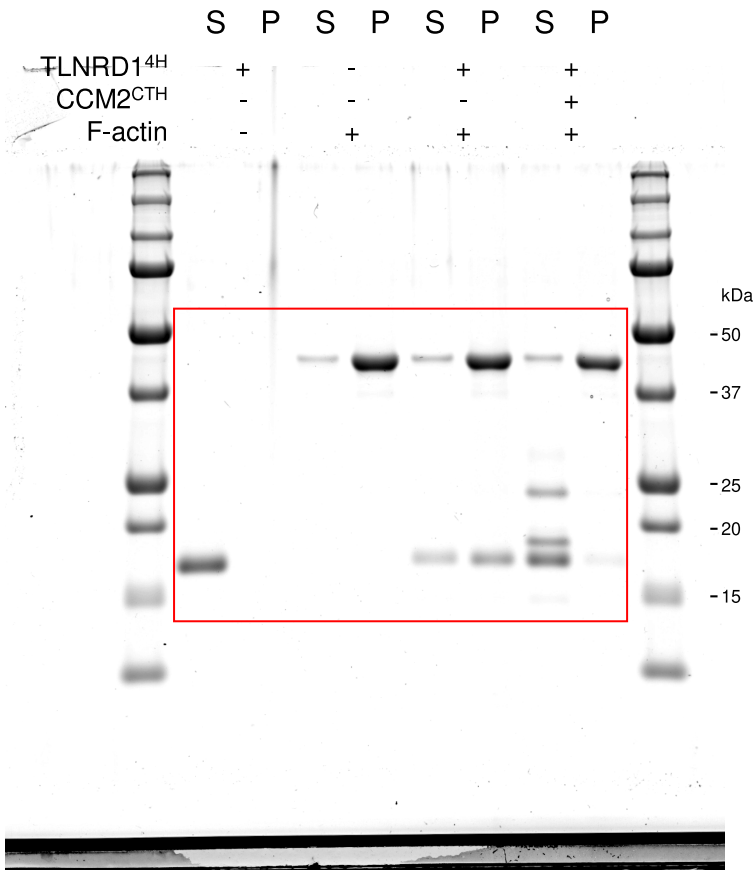

2T

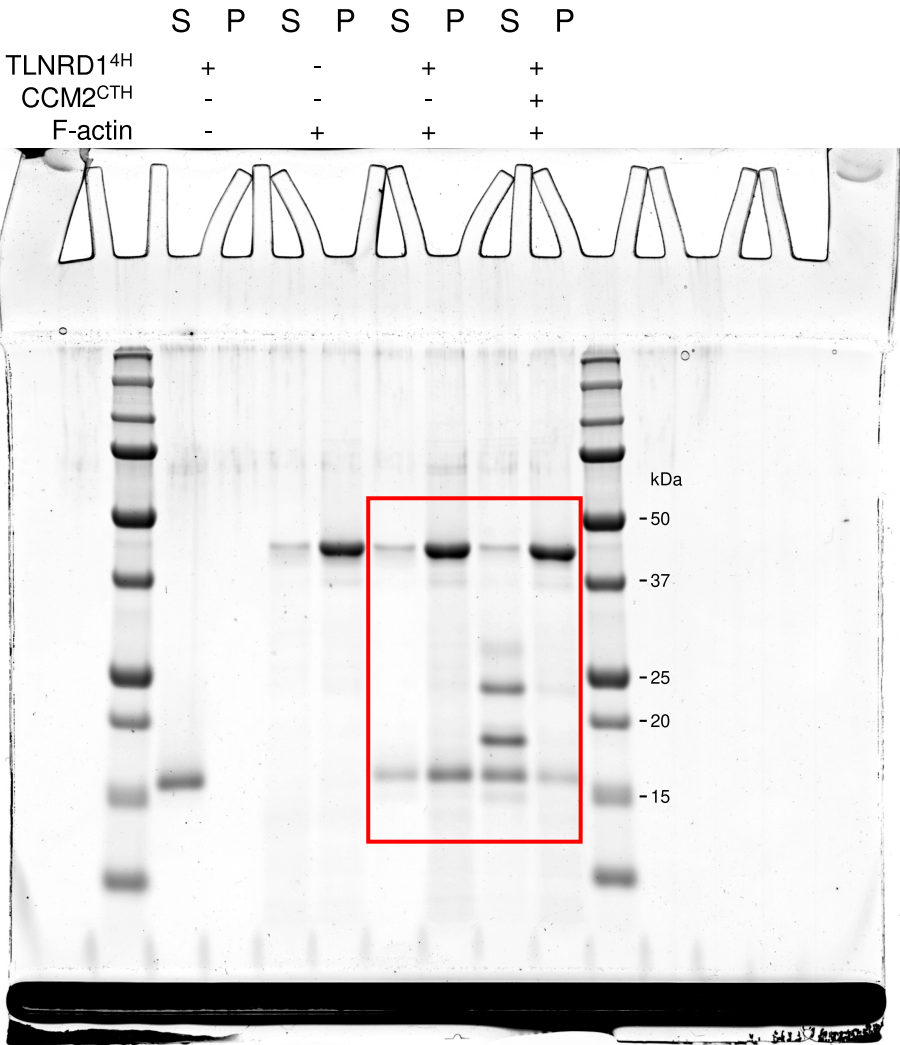

Figure 7D

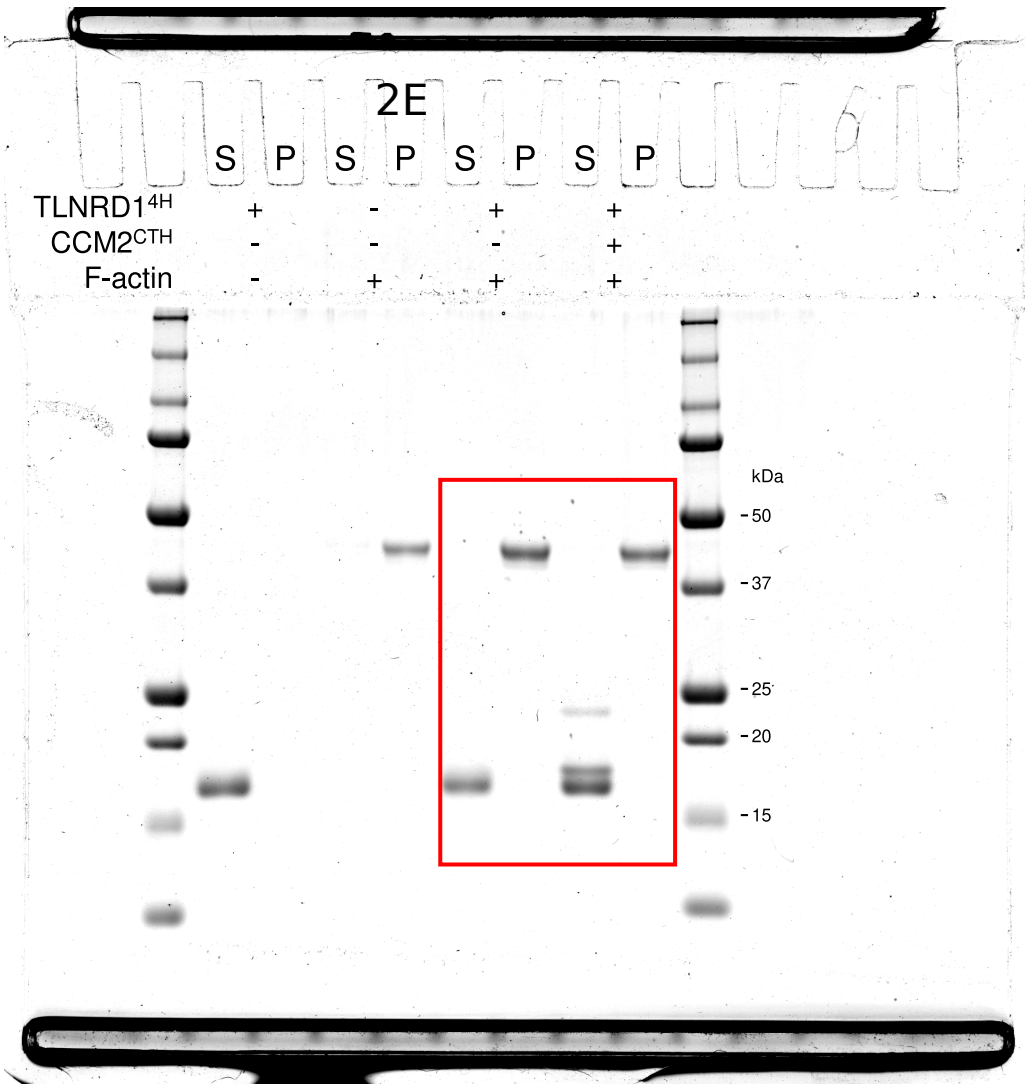

Figure 7E

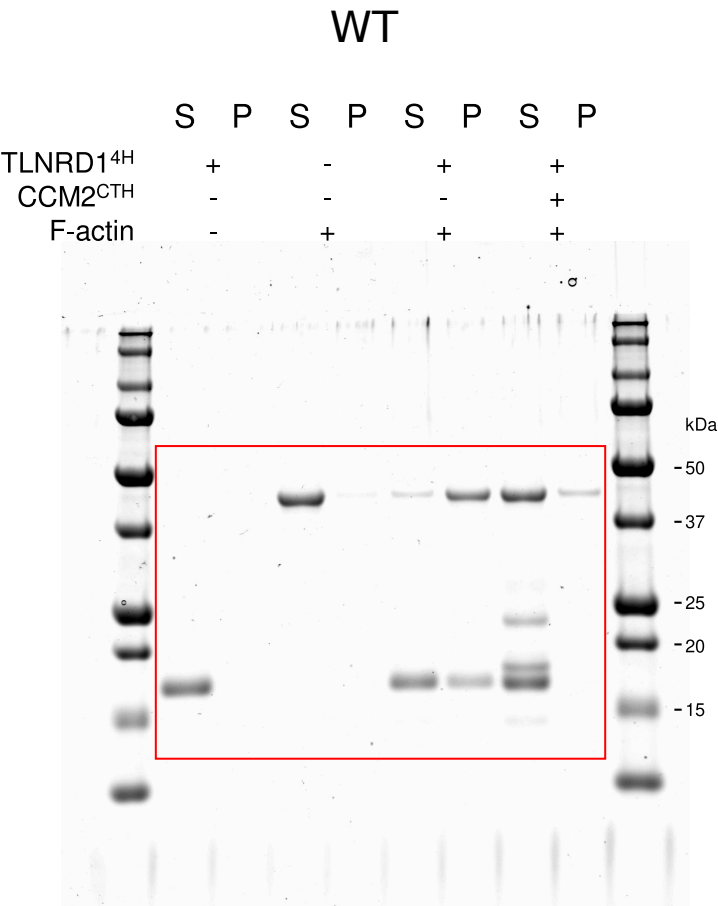

Figure 7E

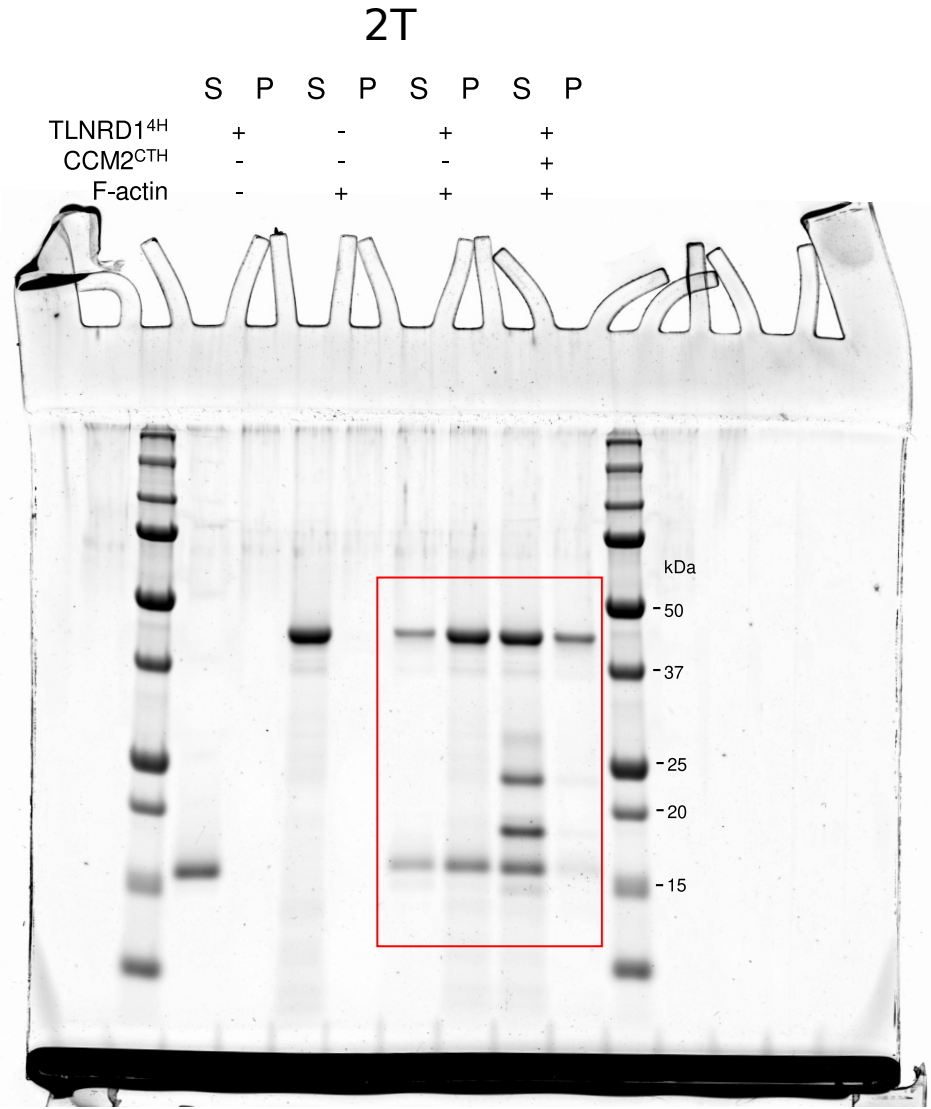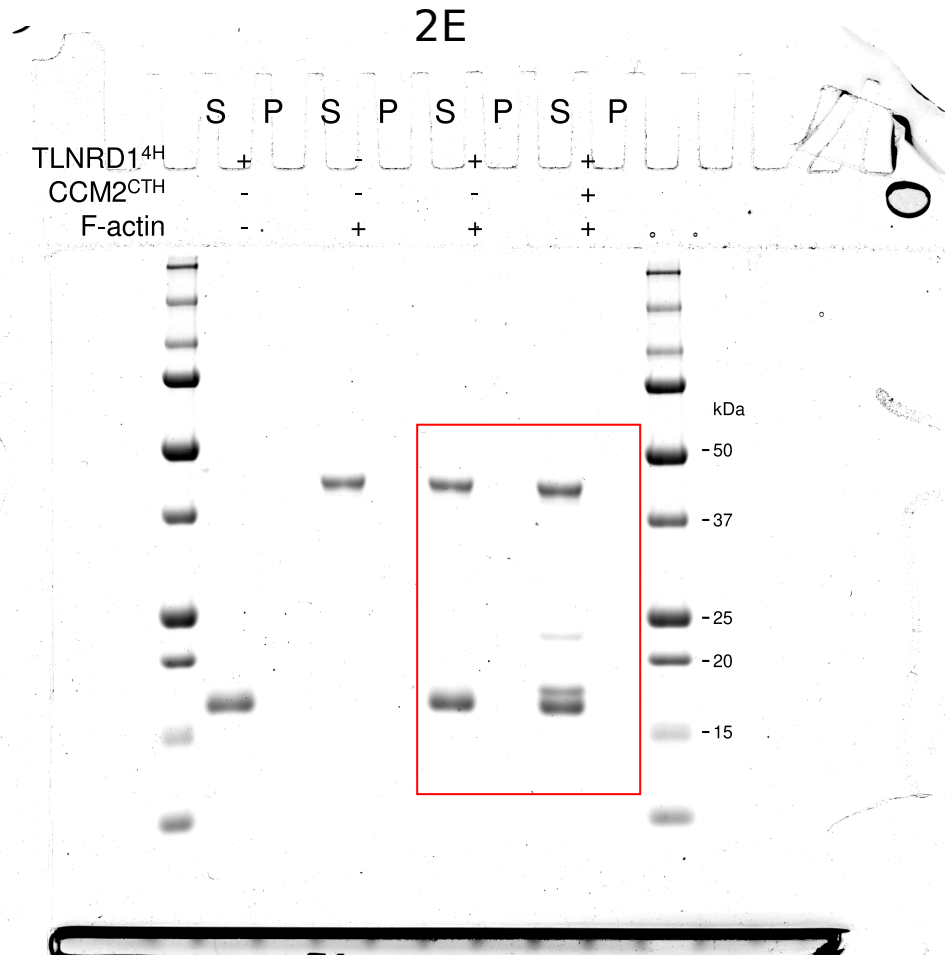

Supplement: SourceData F7 — is the source file for Fig. 7. [file JCB_202310030_SourceDataF7.pdf]

Figure S3A

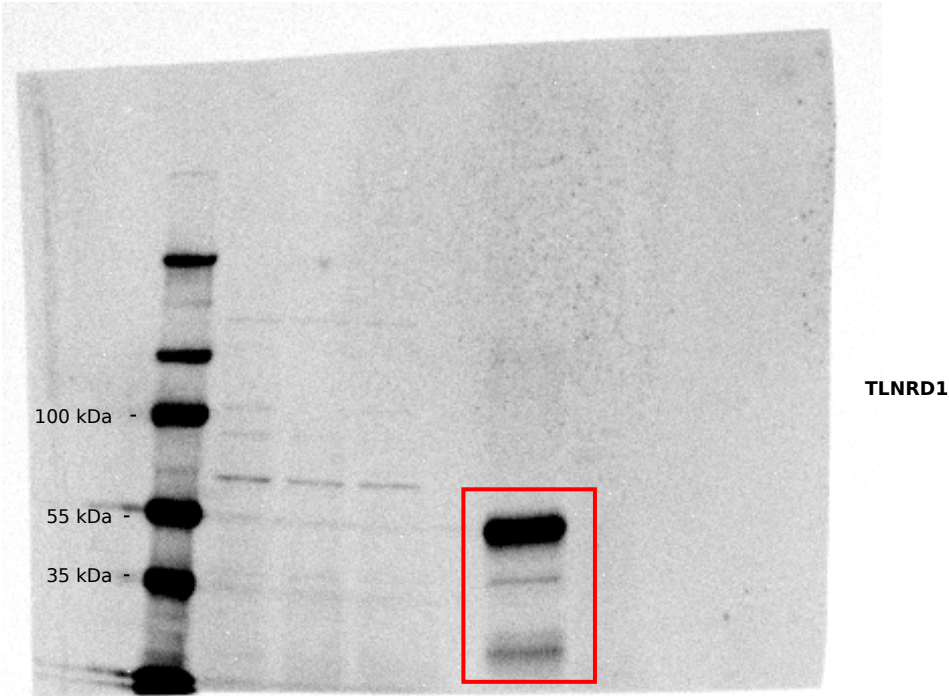

Supplement: SourceData FS3 — is the source file for Fig. S3. [file JCB_202310030_SourceDataFS3.pdf]

Figure S5B

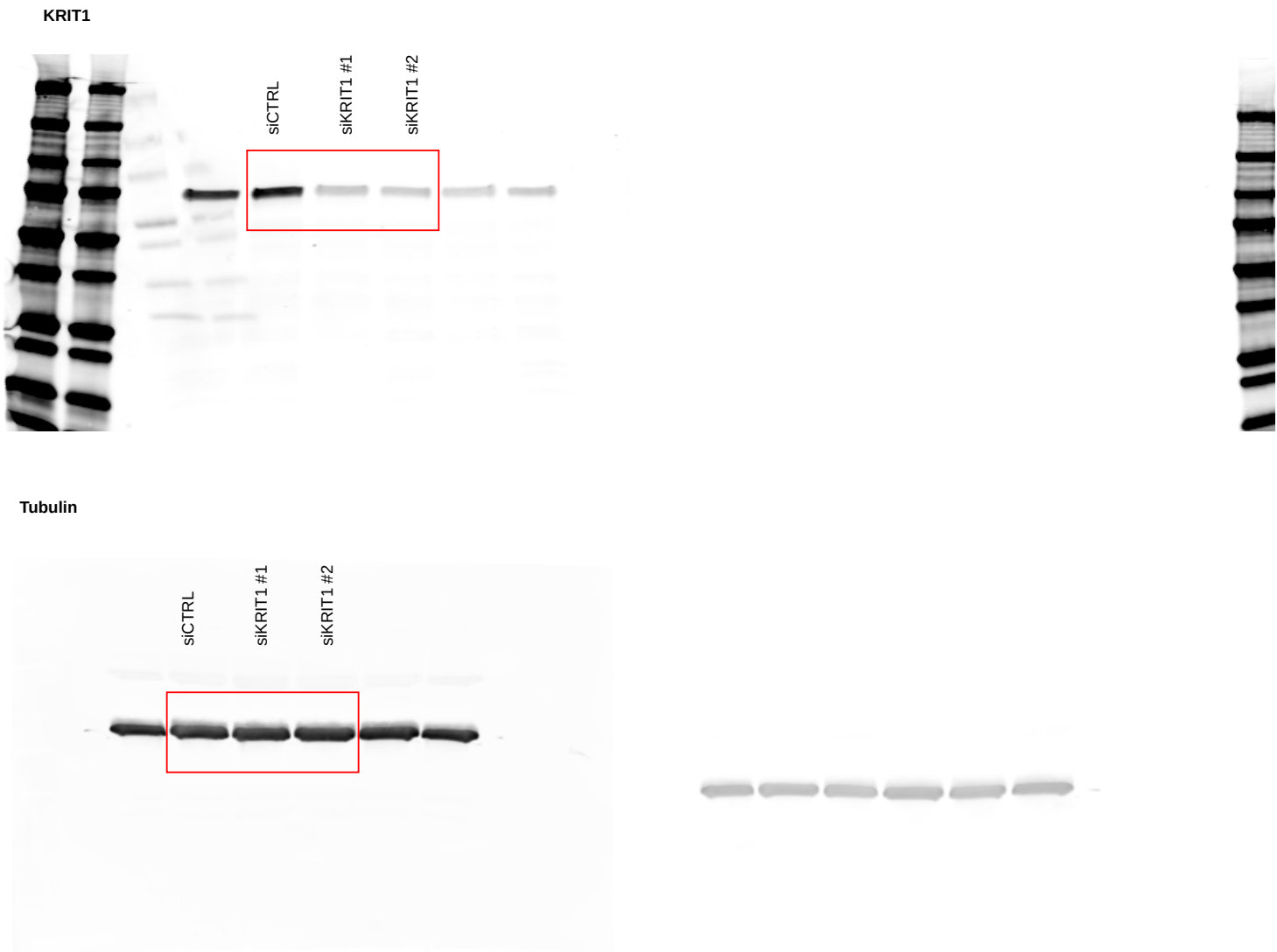

Figure S5D

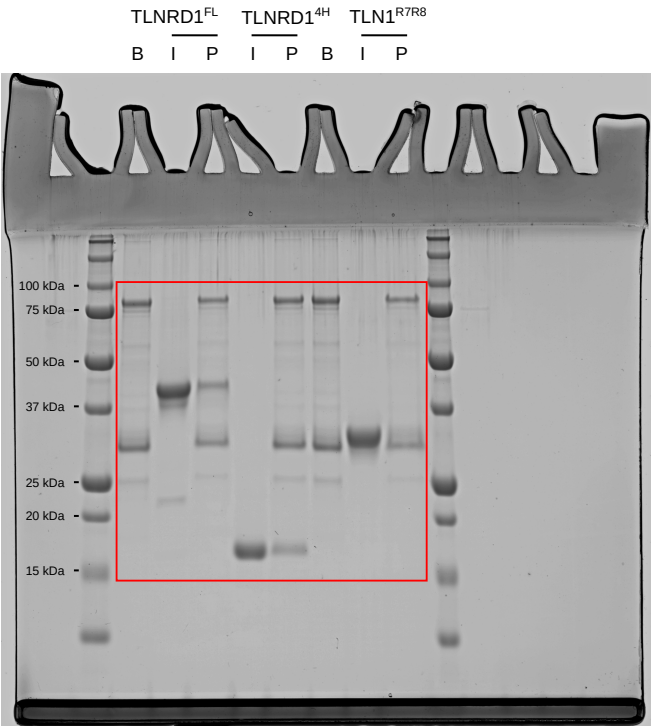

Supplement: SourceData FS5 — is the source file for Fig. S5. [file JCB_202310030_SourceDataFS5.pdf]
